# Effects of GLP-1 receptor agonists on the incidence of contrast-induced acute kidney injury in patients with Type 2 diabetes mellitus undergoing coronary interventions

**DOI:** 10.3389/fendo.2026.1930088

**Published:** 2026-08-27

**Authors:** Dogac Oksen, Muzaffer Aslan, Yunus Emre Yavuz, Cagdas Kaynak, Ebru Serin

**Affiliations:** 1 Department of Cardiology, Altinbas University, Istanbul, Türkiye; 2 Department of Cardiology, Siirt University, Siirt, Türkiye; 3 Department of Cardiology, Sisli Etfal Training and Research Hospital, Istanbul, Türkiye

**Keywords:** contrast-induced acute kidney injury, coronary angiography, GLP-1 receptor agonists, percutaneous coronary intervention, renal protection, type 2 diabetes mellitus

## Abstract

**Background:**

Contrast-induced acute kidney injury (CI-AKI) is a frequent complication of coronary angiography and percutaneous coronary intervention (PCI), particularly in patients with type 2 diabetes mellitus (T2DM). Glucagon-like peptide-1 receptor agonists (GLP-1 RAs) may exert renoprotective effects, but evidence in the setting of contrast exposure remains limited.

**Objective:**

To evaluate the association between GLP-1 RA therapy and CI-AKI in patients with T2DM undergoing coronary angiography or PCI for acute coronary syndromes.

**Methods:**

This retrospective cohort study included 336 patients with T2DM who underwent coronary angiography or PCI between May 2023 and March 2025. Patients receiving stable GLP-1 RA therapy (n=149) were compared with non-users (n=187). Serum creatinine and estimated glomerular filtration rate (eGFR) were measured at baseline and 48–72 hours after the procedure. CI-AKI was defined as a serum creatinine increase of ≥0.5 mg/dL or ≥25% from baseline within 72 hours. Multivariable logistic regression was used to identify independent predictors of CI-AKI.

**Results:**

CI-AKI occurred less frequently among GLP-1 RA users than non-users (8.7% vs 25.7%, p<0.001). At 48–72 hours, eGFR was higher in the GLP-1 RA group (67.1 ± 12.1 vs 58.0 ± 11.8 mL/min/1.73 m², p<0.001), whereas serum creatinine did not differ significantly (1.02 ± 0.73 vs 1.17 ± 1.57 mg/dL, p=0.084). GLP-1 RA use was independently associated with lower odds of CI-AKI (OR 0.290, 95% CI 0.119–0.708; p=0.007). Increasing age (OR 1.332, 95% CI 1.214–1.462; p<0.001) and ST-segment elevation myocardial infarction (OR 5.039, 95% CI 2.368–10.722; p<0.001) were associated with higher odds, whereas higher baseline eGFR was associated with lower odds (OR 0.919, 95% CI 0.862–0.979; p=0.009).

**Conclusion:**

GLP-1 RA therapy was independently associated with a lower risk of CI-AKI in patients with T2DM undergoing contrast-based coronary procedures. These findings suggest a potential renoprotective association but require confirmation in prospective multicenter studies.

## Introduction

Contrast-induced acute kidney injury (CI-AKI) is a significant complication arising from intravascular administration of iodinated contrast media during diagnostic and interventional procedures, notably coronary angiography and percutaneous coronary intervention (PCI). The incidence of CI-AKI varies widely, reported between 1% and 33%, depending on patient risk factors and procedural variables (1, 2). While various definitions for CI-AKI exist, the criteria established by the Kidney Disease: Improving Global Outcomes (KDIGO) initiative are among the most commonly applied. According to KDIGO, CI-AKI is characterized by an increase in serum creatinine of at least 0.5 mg/dL or a relative increase of ≥25% from the baseline value, measured within 72 hours following the exposure (3).

Although CI-AKI is often reversible with appropriate hydration therapy and does not lead to permanent damage, it can result in prolonged hospital and intensive care unit stays. In a subset of patients, it may rarely progress to require temporary or permanent dialysis, and in some cases, may even be associated with increased mortality (4). Numerous studies have investigated the predictors that increase susceptibility to this condition, which contributes significantly to both morbidity and mortality. The likelihood of developing CI-AKI increases in the presence of specific baseline risk factors, including impaired renal function, heart failure with reduced ejection fraction, anemia or blood loss during the procedure, increased contrast media usage, acute myocardial infarction, advanced age, hypotension, and perfusion defects due to systolic dysfunction (5). Among these, diabetes mellitus stands out as one of the most prominent and commonly encountered predictors in routine clinical practice (6). Patients with type 2 diabetes mellitus (T2DM) are particularly susceptible to CI-AKI due to underlying endothelial dysfunction, oxidative stress, and microvascular complications. The presence of T2DM not only increases the risk of CI-AKI but also exacerbates its severity and associated morbidity (7, 8).

Glucagon-like peptide-1 receptor agonists (GLP-1 RAs) are a class of antidiabetic agents that have demonstrated cardiovascular and renal benefits beyond glycemic control. Their mechanisms include anti-inflammatory effects, reduction of oxidative stress, and improvement of endothelial function (9). Clinical studies have shown that GLP-1 RAs significantly reduce the risk of major adverse cardiovascular events (MACE), including myocardial infarction and stroke, in patients with type 2 diabetes mellitus (10). Additionally, these agents have been associated with improvements in renal outcomes, such as slowing the progression of diabetic kidney disease and reducing albuminuria (11).

In the literature, studies involving sodium-glucose cotransporter 2 (SGLT2) inhibitors have demonstrated a significant reduction in the risk of CI-AKI during angiographic procedures (6). In light of these findings, and considering the growing use of GLP-1 RAs in the management of T2DM along with their emerging renal protective properties, it is essential to explore their potential role in reducing the risk of CI-AKI during coronary angiography and PCI due to acute coronary syndromes. This study is therefore designed to assess the impact of GLP-1 RAs on the incidence of CI-AKI in patients with T2DM undergoing coronary interventions.

## Material and methods

### Study design and population

This retrospective cohort study was conducted to evaluate the effects of GLP-1 RAs on the incidence of CI-AKI in patients with T2DM undergoing coronary angiography or PCI due to acute coronary syndromes. The study included patients admitted to our cardiology department between May 2023 and March 2025, who had a confirmed diagnosis of T2DM and underwent contrast-enhanced coronary procedures. Patients were divided into two groups based on their GLP-1 RA usage: those receiving GLP-1 RA therapy for at least 3 months prior to the procedure (GLP-1 group, n=149), and those not receiving GLP-1 RAs (non-GLP-1 group, n=187).

Inclusion criteria for the study encompassed patients aged 18 years or older with a confirmed diagnosis of T2DM who underwent coronary angiography and/or PCI, including those presenting with acute coronary syndromes such as unstable angina, non-ST elevation myocardial infarction (NSTEMI), or ST elevation myocardial infarction (STEMI). Eligible patients were required to have documented serum creatinine levels both at baseline and at 72 hours following contrast exposure to allow for the evaluation of renal function changes.

Exclusion criteria included patients with end-stage renal disease on maintenance dialysis, those with documented acute kidney injury at baseline, individuals who underwent primary PCI in the context of cardiogenic shock or hemodynamic instability, and patients with missing or incomplete follow-up renal function data within the 72-hour post-procedural window. Patients receiving contrast for non-coronary diagnostic procedures were also excluded to ensure procedural homogeneity.

All patients received standard periprocedural hydration protocols in accordance with institutional guidelines to prevent CI-AKI. Baseline demographic, clinical, and laboratory data were collected from electronic medical records. These included age, sex, comorbid conditions, current medications, baseline laboratory values (including creatinine, eGFR, hemoglobin, lipid profile), and procedural characteristics such as contrast volume and hospital stay duration. Serum creatinine and eGFR were measured both before the procedure and at 72 hours following contrast exposure.

In the GLP-1 RA group, patients had been on stable therapy with GLP-1 RAs for a minimum of three months prior to undergoing the coronary procedure. Agents used included commonly prescribed formulations such as liraglutide, dulaglutide, and semaglutide, with dosing regimens based on standard clinical guidelines and individualized according to glycemic control and tolerability. The dosing was consistent with approved therapeutic ranges—liraglutide typically administered at 1.2–1.8 mg daily, dulaglutide at 0.75–1.5 mg weekly, and semaglutide at 0.5–1 mg weekly (12). Treatment adherence was confirmed through patient records and follow-up interviews. No adjustments were made to GLP-1 RA therapy in the immediate preprocedural period.

### Definitions and outcomes

The estimated glomerular filtration rate (eGFR) was calculated using the Levey-modified MDRD equation, which incorporates serum creatinine, age, and sex: 186.3 × (serum creatinine in mg/dL)^–1.154 × (age in years)^–0.203, with a multiplication factor of 0.742 applied for female patients (13).

CI-AKI was defined as either an absolute increase in serum creatinine of ≥0.5 mg/dL or a relative increase of ≥25% from the baseline value, measured within 72 hours following contrast media administration (3).

The primary outcome was the incidence of CI-AKI in the GLP-1 and non-GLP-1 groups. Secondary outcomes included changes in serum creatinine and eGFR levels from baseline to 72 hours post-procedure, length of hospital stay, and identification of independent predictors for CI-AKI.

### Angiography procedure

All coronary angiography and PCI procedures were performed using standard percutaneous femoral or radial arterial access under sterile conditions in a dedicated cardiac catheterization laboratory. Iodinated low-osmolar contrast agents were used and contrast volume was recorded for each patient. All procedures were carried out by experienced interventional cardiologists in accordance with current guidelines. Pre- and post-procedural hydration protocols were applied uniformly to minimize the risk of CI-AKI, typically consisting of isotonic saline at a rate adjusted for renal function and cardiac status. Periprocedural medications, including antiplatelet therapy, anticoagulants, and other cardiovascular drugs, were administered as clinically indicated. Decisions regarding PCI, including stent implantation and lesion selection, were made at the discretion of the operator based on angiographic findings and clinical presentation.

### Statistical analysis

All statistical analyses were performed using IBM SPSS Statistics version 28.0 (IBM Corp., Armonk, NY, USA) and Jamovi version 2.6 (The Jamovi Project, Sydney, Australia). Continuous variables were expressed as mean ± standard deviation (SD) and compared using the Student’s t-test. The Kolmogorov–Smirnov test was used to assess the normality of continuous variables. Categorical variables were presented as frequencies and percentages and compared using the Chi-square test or Fisher’s exact test, as appropriate. Variables with a p-value <0.10 in the univariate analysis together with clinically relevant variables were considered for inclusion in the multivariable binary logistic regression model. Multicollinearity was assessed using the variance inflation factor (VIF), and variables with VIF values >5 were excluded from the final model to improve model stability and avoid unstable coefficient estimates. The final multivariable model included GLP-1 receptor agonist use, age, baseline estimated glomerular filtration rate (eGFR), and ST-segment elevation myocardial infarction (STEMI). Model calibration was evaluated using the Hosmer–Lemeshow goodness-of-fit test. Results were reported as odds ratios (ORs) with corresponding 95% confidence intervals (95% CIs). A two-sided p-value <0.05 was considered statistically significant.

## Results

A total of 440 patients with T2DM who underwent coronary angiography or PCI were initially evaluated for inclusion in the study. After applying the predefined exclusion criteria, 336 patients met the eligibility requirements and were included in the final analysis. Of these, 149 patients were receiving GLP-1 receptor agonist therapy (GLP-1 group), while 187 patients had no history of GLP-1 RA use (non-GLP group).

Baseline demographic and clinical characteristics were largely comparable between the two groups, with no significant differences in age (62.0 ± 7.34 vs. 63.0 ± 6.98 years; p = 0.203), gender distribution (62.4% vs. 62.0% male; p = 0.943), or hypertension prevalence (59.1% vs. 58.8%; p = 0.965). However, patients in the GLP-1 group had significantly lower BMI (23.56 ± 2.16 vs. 24.29 ± 2.82 kg/m²; p = 0.009), glucose levels (177.56 ± 37.50 vs. 190.79 ± 49.52 mg/dL; p = 0.007), and urea concentrations (38.79 ± 7.10 vs. 40.96 ± 11.40 mg/dL; p = 0.042) compared to the non-GLP group (**Table 1**).

**Table 1:** Baseline demographic, clinical, and laboratory characteristics of GLP-1 receptor agonist users and non-users.

Variable	GLP 1 (n: 149)	NON GLP (n: 187)	P value
Age, years	62.0 ± 7.34	63.0 ± 6.98	0.203
Gender, Male, %(n)	62.4 (93)	62(116)	0.943
Hypertension, %(n)	59.1 (88)	58.8 (110)	0.965
BMI, kg/m2	23.56 ± 2.16	24.29 ± 2.82	0.009
Glucose, mg/dL	177.56 ± 37.50	190.79 ± 49.52	0.007
Urea, mg/dL	38.79 ± 7.10	40.96 ± 11.40	0.042
Creatinine (basal), mg/dL	1.0 ±0.60	0.97 ± 0.72	0.102
Creatinine (72th hours), mg/dL	1.02 ± 0.73	1.17 ± 1.57	0.084
eGFR basal, mL/min/1.73m2	68.7 ± 11.8	70.8 ± 11.1	0.092
eGFR 72th hour, mL/min/1.73m2	67.1 ± 12.1	58.0 ± 11.8	<0.001
CI- AKI	8.7 (13)	25.7 (48)	<0.001
LVEF, %	60.99 ± 3.50	61.39 ± 3.14	0.271
Hemoglobin, g/dL	13.55 ± 0.99	12.99 ± 0.81	<0.001
WBC,	11.09 ± 1.53	11.29 ± 1.57	0.241
Platelet,	249.25 ± 46.11	257.80 ± 46.11	0.083
Total Cholesterol, mg/dL	192.69 ± 37.82	200.84 ± 41.27	0.063
LDL- C, mg/dL	121.72 ± 24.42	116.92 ± 24.42	0.074
HDL- C, mg/dL	41.18 ± 5.90	41.04 ± 7.00	0.855
Triglyceride, mg/dL	189.82 ± 76.34	174.56 ± 84.55	0.087
HbA1c, %	7.58 ± 1.39	7.70 ± 1.68	0.493

GLP-1, glucagon-like peptide-1; BMI, body mass index; eGFR, estimated glomerular filtration rate; CI-AKI, contrast-induced acute kidney injury; LVEF, left ventricular ejection fraction; WBC, white blood cell count; LDL-C, low-density lipoprotein cholesterol; HDL-C, high-density lipoprotein cholesterol; HbA1c, glycated hemoglobin; SD, standard deviation.

The incidence of CI-AKI was markedly lower in the GLP-1 group (8.7%, n = 13) compared to the non-GLP group (25.7%, n = 48; p < 0.001). At baseline, renal function was comparable between the two groups, with no significant differences in serum creatinine (1.00 ± 0.60 vs. 0.97 ± 0.72 mg/dL, p = 0.102) or estimated glomerular filtration rate (eGFR) (68.7 ± 11.8 vs. 70.8 ± 11.1 mL/min/1.73 m², p = 0.092) (**Table 1**). At 48–72 hours after contrast exposure, the GLP-1 RA group demonstrated significantly better preservation of renal function, with higher eGFR values than the non-GLP-1 RA group (67.1 ± 12.1 vs. 58.0 ± 11.8 mL/min/1.73 m², p < 0.001), whereas the between-group difference in serum creatinine did not reach statistical significance (1.02 ± 0.73 vs. 1.17 ± 1.57 mg/dL, p = 0.084). Left ventricular ejection fraction (LVEF) did not differ significantly between the groups (60.99 ± 3.50% vs. 61.39 ± 3.14%; p = 0.271). Hemoglobin levels were higher in the GLP-1 group (13.55 ± 0.99 vs. 12.99 ± 0.81 g/dL; p < 0.001), while other laboratory parameters including white blood cell count, platelet count, lipid profile, and HbA1c showed no significant differences (**Table 1**).

To further characterize patients according to CI-AKI status, additional comparisons of continuous and categorical clinical characteristics were performed. Patients who developed CI-AKI were generally older and exhibited a less favorable metabolic and renal profile, including lower baseline and post-procedural eGFR values, higher glucose levels, greater contrast volume exposure, and a higher prevalence of STEMI. Detailed results are presented in **Supplementary Tables 1**, **2**.

Medication profiles were comparable between the two groups, with no statistically significant differences in the use of ACE inhibitors, ARBs, beta-blockers, calcium channel blockers, diuretics, statins, metformin, insulin, or SGLT-2 inhibitors (**Table 2**).

**Table 2:** Comparison of baseline medical treatments between GLP-1 receptor agonist users and non-users.

Variable	GLP 1	NON GLP	P value
ACE- inhibitors, %(n)	40.9 (61)	38.2 (71)	0.606
ARB, %(n)	17.4 (26)	18.7 (35)	0.765
Beta-blokers, %(n)	50.3 (75)	48.7 (91)	0.761
Ca-Channel blokers, %(n)	16.8 (25)	23.5 (44)	0.128
Diuretics, %(n)	27.5 (41)	24.6 (46)	0.544
Statins, %(n)	39.6 (59)	36.4 (68)	0.544
Metformin, %(n)	61.7 (92)	63.1 (118)	0.799
Insulin, %(n)	28.9 (43)	33.7 (63)	0.344
Thiazolidinedion, %(n)	9.4 (14)	10.7 (20)	0.695
Sulfanilurea, %(n)	16.1 (24)	18.7 (35)	0.532
DPP4 inhibitors, %(n)	45 (67)	46 (86)	0.852
SGLT-2, %(n)	20.8 (31)	21.4 (40)	0.896
GLP-1, % (n)	100(149)	None	-

ACE, angiotensin-converting enzyme; ARB, angiotensin II receptor blocker; Ca-channel, calcium channel; DPP-4, dipeptidyl peptidase-4; SGLT-2, sodium-glucose cotransporter-2; GLP-1, glucagon-like peptide-1.

Regarding presentation and procedural characteristics, the frequency of unstable angina pectoris (USAP), non-ST elevation myocardial infarction (NSTEMI) and ST elevation myocardial infarction (STEMI) were similar between groups. Although PCI was performed less frequently in the GLP-1 group than in the non-GLP group, this difference did not reach statistical significance (59.06% vs. 65.2%; p = 0.054). The mean contrast volume was also comparable between groups (159.92 ± 46.60 vs. 161.73 ± 39.00 mL; p = 0.698). Interestingly, length of hospital stay was longer in GLP-1 users (2.99 ± 1.41 vs. 2.49 ± 1.12 days; p < 0.001), potentially reflecting more frequent PCI interventions (**Table 3**).

**Table 3:** Clinical presentation and procedural characteristics of GLP-1 receptor agonist users and non-users.

Variable	GLP 1 (n: 149)	NON GLP (n: 187)	P value
USAP, %(n)	33.5 (50)	36.8 (30)	0.525
NON-STEMI, %(n)	23.5 (35)	26.7 (50)	0.496
STEMI, %(n)	43 (64)	36.4 (68)	0.219
PCI, %(n)	59.06 (88)	65.2 (122)	0.054
Contrast volume, mL	159.92 ± 46.60	161.73 ± 39	0.698
Length of Hospital Stay, days	2.99 ± 1.41	2.49 ± 1.12	<0.001

USAP, unstable angina pectoris; NSTEMI, non–ST-elevation myocardial infarction; STEMI, ST-elevation myocardial infarction; PCI, percutaneous coronary intervention; GLP-1, glucagon-like peptide-1; SD, standard deviation.

**Figure 1:**
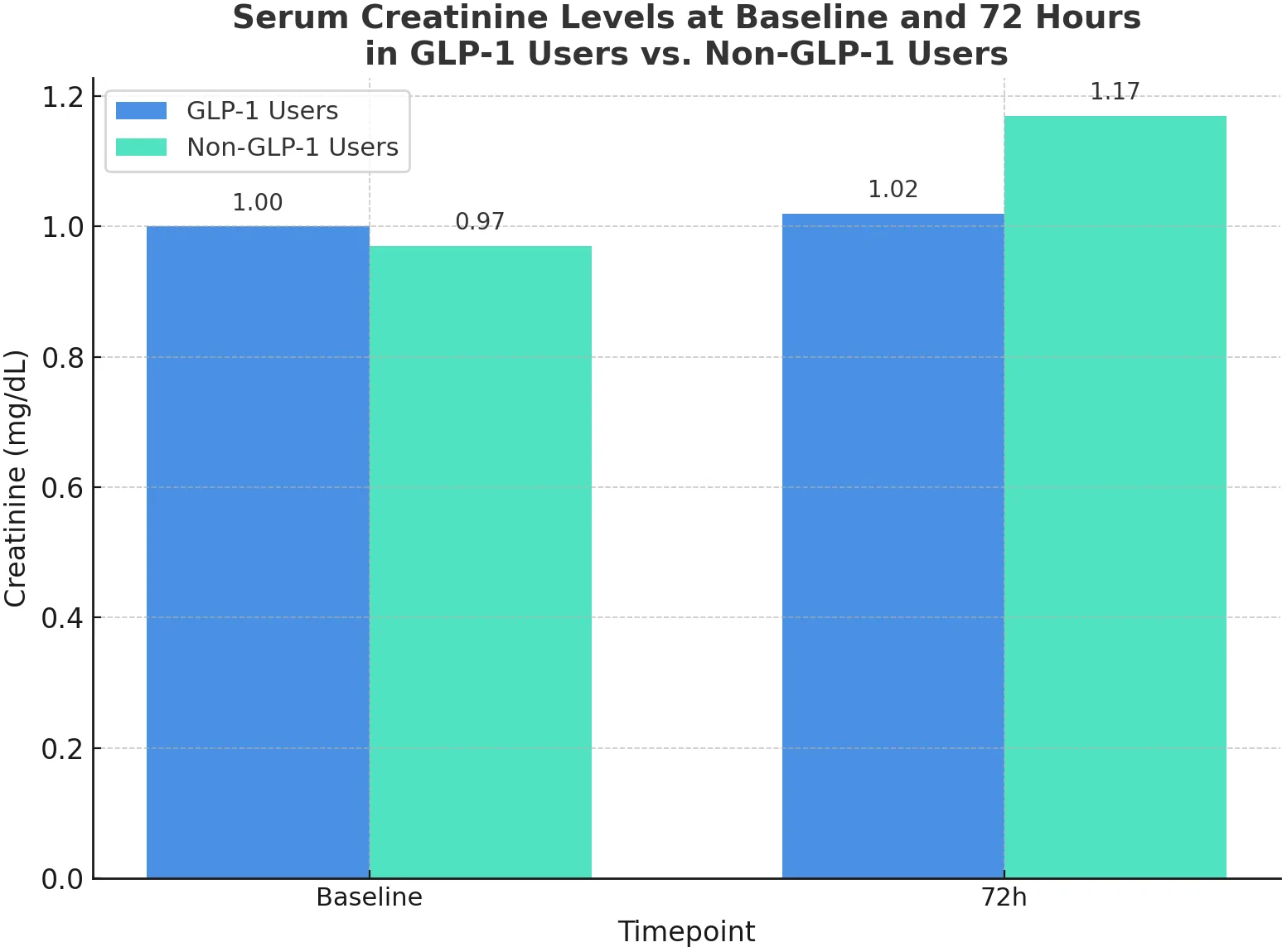
Comparison of serum creatinine levels at baseline and 72 hours following contrast exposure in GLP-1 receptor agonist users and non-users. Patients receiving GLP-1 therapy demonstrated significantly lower serum creatinine levels at 72 hours compared to non-users. Bars represent mean creatinine values (mg/dL) with numerical values indicated above each bar. GLP-1 receptor agonist usage was associated with attenuation of contrast-associated renal dysfunction.

Within-group analysis showed no significant changes in serum creatinine (p = 0.214) or eGFR (p = 0.128) among GLP-1 RA users, whereas non-users exhibited a significant increase in serum creatinine and a significant decline in eGFR over the same period (both p ≤ 0.001) (**Table 4** and **Figure 1**).

**Table 4:** Comparison of baseline and 72-hour serum creatinine and estimated glomerular filtration rate between GLP-1 receptor agonist users and non-users.

Variable	Basal creatinine	72th hour creatinine	P value
GLP- 1 user group	1.0± 0.60	1.02 ± 0.73	0.214
Non-user group	0.97 ± 0.07	1.17 ± 1.57	<0.001
	**Basal eGFR**	**72th hour eGFR**	
GLP- 1 user group	68.7 ± 11.8	67.1 ± 12.1	0.128
Non-user group	70.8 ± 11.1	58.0 ± 11.8	0.001

eGFR, estimated glomerular filtration rate; GLP-1, glucagon-like peptide-1; CI-AKI, contrast-induced acute kidney injury; SD, standard deviation.

In the multivariable logistic regression analysis, GLP-1 RA use was independently associated with a significantly lower risk of CI-AKI (OR: 0.290; 95% CI: 0.119–0.708; p = 0.007). Other independent predictors included increasing age (OR: 1.332; 95% CI: 1.214–1.462; p < 0.001), baseline estimated glomerular filtration rate (eGFR) (OR: 0.919; 95% CI: 0.862–0.979; p = 0.009), and ST-segment elevation myocardial infarction (STEMI) (OR: 5.039; 95% CI: 2.368–10.722; p < 0.001) (**Table 5**).

**Table 5:** Independent predictors of contrast-associated acute kidney injury: binary logistic regression analysis.

Variable	OR	Confidence interval	P value
Age	1.33	1.214 – 1.462	<0.001
GLP-1 usage	0.290	0.119 – 0.708	0.007
Basal eGFR	0.919	0.862 – 0.979	0.009
STEMI	5.039	2.368 – 10.722	<0.001

OR, odds ratio; CI, confidence interval; GLP-1, glucagon-like peptide-1; eGFR, estimated glomerular filtration rate; PCI, percutaneous coronary intervention; STEMI, ST-segment elevation myocardial infarction.

## Discussion

This study observed that the use of GLP-1 RAs in patients with T2DM undergoing coronary angiography or PCI was associated with a significantly lower incidence of CI-AKI. Specifically, the incidence of CI-AKI in the GLP-1 group was 8.7%, compared to 25.7% in the non-GLP group (p < 0.001), suggesting a potentially protective effect of GLP-1 RAs on renal function following contrast exposure. Furthermore, the increase in serum creatinine and the decrease in eGFR were also less pronounced in the GLP-1 group, suggesting a protective effect on renal function.

Beyond their established metabolic benefits in T2DM, GLP-1 RAs have recently emerged as promising agents with renoprotective properties. A growing body of evidence suggests that GLP-1 RAs can positively influence kidney outcomes through both direct renal mechanisms and indirect systemic effects. GLP-1 RAs improve several traditional risk factors for chronic kidney disease (CKD), including hyperglycemia, hypertension, dyslipidemia, and obesity. These systemic improvements indirectly contribute to renal protection. Additionally, a natriuretic effect associated with GLP-1 RA use may help reduce intravascular volume and blood pressure, thus attenuating pressure-related renal injury (14).

More importantly, a range of direct renal effects has also been described. GLP-1 receptors are expressed in renal tubular cells, and activation of these receptors has been shown to reduce oxidative stress and inflammation—two key contributors to the progression of diabetic nephropathy (15, 16). Furthermore, these agents may reduce glomerular hyperfiltration, helping to preserve renal structural integrity and delay nephron loss (17). These mechanisms are particularly relevant in the setting of contrast exposure, where oxidative injury and endothelial dysfunction play central roles in the pathogenesis of CI-AKI.

Our results align with major cardiovascular outcome trials (CVOTs) that have explored the renal effects of GLP-1 RAs. In the LEADER trial, liraglutide significantly reduced the risk of nephropathy, primarily by decreasing the development of macroalbuminuria (18). Similarly, the SUSTAIN-6 trial showed that semaglutide lowered the risk of new or worsening nephropathy by 36%, while the REWIND trial demonstrated a protective effect of dulaglutide on composite renal outcomes even in patients without overt nephropathy (19, 20). These findings suggest a class effect and underscore the clinical relevance of our observations.

While most of the evidence to date has focused on long-term renal outcomes in patients with diabetic kidney disease, our study adds to the literature by emphasizing the short-term renal protective potential of GLP-1 RAs in the context of contrast exposure—a setting characterized by acute tubular injury and oxidative stress. The observed reduction in CI-AKI in GLP-1 users suggests a clinically meaningful benefit that may warrant consideration in high-risk diabetic patients undergoing coronary interventions.

These findings align with and expand upon the existing body of research that has predominantly focused on the renoprotective effects of SGLT2 inhibitors. A recent study by Cabuk et al. demonstrated that SGLT2 inhibitor use in a similar patient population reduced the incidence of CI-AKI from 26.4% to 9.0%, a reduction that closely mirrors our results with GLP-1 Ras (6). Both studies show that modern antidiabetic agents may exert organ-protective effects beyond glycemic control, though the underlying mechanisms may differ.

Our findings align with prior studies investigating the impact of SGLT2 inhibitors on CI-AKI. For instance, Hao et al. conducted a propensity-matched analysis demonstrating that SGLT2 inhibitor use was associated with a 63% reduction in the odds of developing CI-AKI among patients with T2DM undergoing PCI (21). Similarly, Paolisso et al. reported that diabetic patients with acute myocardial infarction treated with SGLT2 inhibitors had a significantly lower incidence of CI-AKI compared to non-users, with an OR of 0.356 (22).

Interestingly, in both our study and Cabuk et al.’s, patients receiving modern antidiabetic therapies had slightly longer hospital stays despite reduced CI-AKI incidence (6). This paradox may be explained by a higher rate of PCI in these groups, necessitating prolonged monitoring, rather than a reflection of renal complications per se.

Unlike many studies that exclude patients with preserved renal function, our study demonstrates that even in patients with baseline eGFR values above 60 mL/min/1.73 m², GLP-1 RAs confer renal protection. This broadens the potential applicability of these agents beyond the traditionally “at-risk” population and underscores the importance of early and sustained GLP-1 RA therapy in diabetic cardiovascular patients.

Furthermore, these benefits appear to be independent of glycemic control, as previously reported in CVOTs, where the renal advantages persisted after adjusting for changes in HbA1c, weight, and blood pressure (23, 24). This supports the hypothesis that GLP-1 RAs have intrinsic renoprotective actions that may be particularly beneficial in patients undergoing procedures associated with acute renal stress.

The independent association between STEMI and contrast-associated kidney injury observed in our cohort is biologically plausible. Unlike elective coronary procedures, primary PCI for STEMI is performed urgently, often precluding adequate intravenous hydration before contrast exposure. Similarly, a recent study in patients with acute coronary syndrome demonstrated that impaired baseline renal function and hemodynamic compromise were important determinants of CI-AKI, emphasizing the contribution of renal hypoperfusion to post-procedural kidney injury (25). These findings support our observation that STEMI, a clinical setting frequently accompanied by greater hemodynamic instability, was independently associated with an increased risk of CI-AKI. In addition, acute hemodynamic compromise, heightened inflammatory response, and larger infarct size may further increase renal susceptibility to ischemic and contrast-mediated injury, explaining the higher incidence of kidney injury reported in STEMI patients (26, 27).

## Limitations

This real-world observational study has several limitations. First, its retrospective, single-center design precludes the establishment of causal relationships and may limit the generalizability of the findings. Although multivariable logistic regression was performed to adjust for clinically relevant confounding factors, residual confounding from unmeasured variables cannot be completely excluded. Furthermore, propensity score matching or inverse probability weighting was not performed; therefore, the potential influence of selection bias should be considered when interpreting our results. To improve the robustness of the multivariable model, multicollinearity was systematically assessed using the variance inflation factor (VIF), and variables demonstrating significant collinearity were excluded from the final model. Nevertheless, despite these statistical adjustments, residual confounding remains possible. In addition, we did not perform subgroup analyses according to individual GLP-1 receptor agonists (e.g., liraglutide versus semaglutide), which may differ in their pharmacological potency and potential renoprotective effects. The follow-up period was limited to 72 hours after contrast exposure and therefore may not have captured delayed or persistent renal dysfunction. Furthermore, biomarkers of tubular injury, such as neutrophil gelatinase-associated lipocalin (NGAL) or kidney injury molecule-1 (KIM-1), were not available, which may have limited the detection of subclinical kidney injury. Finally, although hydration protocols and contrast volumes were comparable between groups, the potential influence of unmeasured procedural and hemodynamic factors cannot be entirely excluded. Therefore, prospective multicenter studies with propensity score-based analyses or randomized controlled designs are warranted to validate our findings.

## Conclusion

In conclusion, GLP-1 receptor agonist therapy was independently associated with a lower risk of contrast-induced acute kidney injury in patients with type 2 diabetes mellitus undergoing contrast-based coronary interventions. Increasing age and ST-segment elevation myocardial infarction (STEMI) were identified as independent predictors of CI-AKI, whereas higher baseline estimated glomerular filtration rate (eGFR) was independently associated with a lower risk of CI-AKI. These findings support the potential renoprotective role of GLP-1 receptor agonists in high-risk cardiovascular patients. However, given the retrospective observational design and the possibility of residual confounding, the findings should be interpreted with caution. Prospective, multicenter randomized controlled studies are warranted to confirm these findings and to further elucidate the underlying mechanisms.

## Data Availability

The raw data supporting the conclusions of this article will be made available by the authors, without undue reservation.
