# Supplementary material for: Effects of GLP-1 receptor agonists on the incidence of contrast-induced acute kidney injury in patients with Type 2 diabetes mellitus undergoing coronary interventions
Source: Front Endocrinol (Lausanne). 2026 Aug 27;17:1930088. doi: 10.3389/fendo.2026.1930088 (PMC13557935; doi:10.3389/fendo.2026.1930088)
Supplement: Supplementary file 1 [file Table1.docx]

| **Supplementary Table S1.** Comparison of baseline characteristics between patients with and without contrast-induced acute kidney injury (CI-AKI). | | | |
| --- | --- | --- | --- |
| Variable | No CI-AKI (n = 275) | CI-AKI (n = 61) | p- value |
| Age, years | 61.1 ± 6.9 | 69.2 ± 3.6 | <0.001 |
| BMI, kg/m² | 23.69 ± 2.74 | 25.23 ± 0.86 | <0.001 |
| Glucose, mg/dL | 181.1 ± 48.5 | 202.1 ± 14.9 | <0.001 |
| Urea, mg/dL | 39.2 ± 10.5 | 43.7 ± 3.2 | 0.001 |
| Baseline creatinine, mg/dL | 0.983 ± 0.076 | 0.998 ± 0.018 | 0.140 |
| Creatinine (48–72 h), mg/dL | 1.089 ± 0.151 | 1.182 ± 0.084 | <0.001 |
| Baseline eGFR, mL/min/1.73 m² | 70.8 ± 11.6 | 65.5 ± 9.7 | <0.001 |
| eGFR (48–72 h), mL/min/1.73 m² | 63.8 ± 12.8 | 54.1 ± 8.9 | <0.001 |
| LVEF, % | 61.0 ± 3.6 | 62.2 ± 0.8 | 0.008 |
| Hemoglobin, g/dL | 13.22 ± 1.03 | 13.36 ± 0.27 | 0.303 |
| White blood cell count, ×10⁹/L | 11.08 ± 1.69 | 11.79 ± 0.36 | 0.001 |
| Platelet count, ×10³/µL | 250.7 ± 48.6 | 269.1 ± 12.9 | 0.003 |
| Total cholesterol, mg/dL | 194.2 ± 43.2 | 211.0 ± 11.5 | 0.003 |
| LDL cholesterol, mg/dL | 117.7 ± 26.8 | 125.1 ± 5.7 | 0.033 |
| HDL cholesterol, mg/dL | 40.7 ± 7.1 | 43.0 ± 1.7 | 0.012 |
| Triglycerides, mg/dL | 176.7 ± 88.7 | 202.2 ± 19.4 | 0.027 |
| HbA1c, % | 7.55 ± 1.70 | 8.11 ± 0.38 | 0.010 |
| Contrast volume, mL | 156.4 ± 45.4 | 181.3 ± 13.0 | <0.001 |
| Data are presented as mean ± standard deviation (SD). Continuous variables were compared using the independent samples Student's t-test. BMI, body mass index; eGFR, estimated glomerular filtration rate; LVEF, left ventricular ejection fraction; LDL, low-density lipoprotein; HDL, high-density lipoprotein; HbA1c, glycated hemoglobin; CI-AKI, contrast-induced acute kidney injury. | | | |

| **Supplementary Table S2.** Comparison of categorical clinical characteristics between patients with and without contrast-induced acute kidney injury (CI-AKI). | | | |
| --- | --- | --- | --- |
| Variable | No CI-AKI (n=275) | CI-AKI (n=61) | p-value |
| GLP-1 RA use, n (%) | 136 (49.5) | 13 (21.3) | <0.001 |
| Female sex, n (%) | 176 (64.0) | 33 (54.1) | 0.149 |
| Hypertension, n (%) | 157 (57.1) | 41 (67.2) | 0.146 |
| ACE inhibitor use, n (%) | 101 (36.9) | 31 (50.8) | 0.044 |
| ARB use, n (%) | 53 (19.3) | 8 (13.1) | 0.259 |
| Beta-blocker use, n (%) | 133 (48.4) | 33 (54.1) | 0.418 |
| Calcium channel blocker use, n (%) | 51 (18.5) | 18 (29.5) | 0.055 |
| Diuretic use, n (%) | 63 (22.9) | 24 (39.3) | 0.008 |
| Statin use, n (%) | 90 (32.7) | 37 (60.7) | <0.001 |
| Metformin use, n (%) | 167 (60.7) | 43 (70.5) | 0.154 |
| Insulin use, n (%) | 102 (37.1) | 25 (41) | 0.570 |
| Thiazolidinedione use, n (%) | 28 (10.2) | 6 (9.8) | 0.935 |
| Sulfonylurea use, n (%) | 50 (18.2) | 9 (14.8) | 0.524 |
| Unstable angina, n (%) | 31 (11.3) | 9 (14.8) | 0.448 |
| NSTEMI, n (%) | 33 (12.0) | 52 (85.2) | <0.001 |
| STEMI, n (%) | 87 (31.6) | 45 (73.8) | <0.001 |
| Abbreviations: GLP-1 RA, glucagon-like peptide-1 receptor agonist; ACE, angiotensin-converting enzyme; ARB, angiotensin II receptor blocker; NSTEMI, non-ST-segment elevation myocardial infarction; STEMI, ST-segment elevation myocardial infarction; CI-AKI, contrast-induced acute kidney injury. | | | |
